# Understanding the population consequences of disturbance

**DOI:** 10.1002/ece3.4458

**Published:** 2018-09-12

**Authors:** Enrico Pirotta, Cormac G. Booth, Daniel P. Costa, Erica Fleishman, Scott D. Kraus, David Lusseau, David Moretti, Leslie F. New, Robert S. Schick, Lisa K. Schwarz, Samantha E. Simmons, Len Thomas, Peter L. Tyack, Michael J. Weise, Randall S. Wells, John Harwood

**Affiliations:** ^1^ Department of Mathematics and Statistics Washington State University Vancouver Washington; ^2^ School of Biological, Earth and Environmental Sciences University College Cork Cork Ireland; ^3^ SMRU Consulting New Technology Centre St Andrews UK; ^4^ Department of Ecology and Evolutionary Biology University of California Santa Cruz California; ^5^ Department of Environmental Science and Policy University of California Davis California; ^6^ Department of Fish, Wildlife and Conservation Biology Colorado State University Fort Collins Colorado; ^7^ Anderson‐Cabot Center for Ocean Life New England Aquarium Boston Massachusetts; ^8^ School of Biological Sciences University of Aberdeen Aberdeen UK; ^9^ Naval Undersea Warfare Center Newport Rhode Island; ^10^ Duke University Durham North Carolina; ^11^ Centre for Research into Ecological and Environmental Modelling University of St Andrews St Andrews UK; ^12^ Institute of Marine Sciences University of California Santa Cruz California; ^13^ Marine Mammal Commission Bethesda Maryland; ^14^ Sea Mammal Research Unit Scottish Oceans Institute School of Biology University of St Andrews St Andrews UK; ^15^ Office of Naval Research Marine Mammal & Biology Program Arlington Virginia; ^16^ Chicago Zoological Society's Sarasota Dolphin Research Program c/o Mote Marine Laboratory Sarasota Florida

**Keywords:** anthropogenic disturbance, environmental impact assessments, marine mammals, nonconsumptive effects, population consequences, trait‐mediated indirect interactions, uncertainty

## Abstract

Managing the nonlethal effects of disturbance on wildlife populations has been a long‐term goal for decision makers, managers, and ecologists, and assessment of these effects is currently required by European Union and United States legislation. However, robust assessment of these effects is challenging. The management of human activities that have nonlethal effects on wildlife is a specific example of a fundamental ecological problem: how to understand the population‐level consequences of changes in the behavior or physiology of individual animals that are caused by external stressors. In this study, we review recent applications of a conceptual framework for assessing and predicting these consequences for marine mammal populations. We explore the range of models that can be used to formalize the approach and we identify critical research gaps. We also provide a decision tree that can be used to select the most appropriate model structure given the available data. *Synthesis and applications:* The implementation of this framework has moved the focus of discussion of the management of nonlethal disturbances on marine mammal populations away from a rhetorical debate about defining negligible impact and toward a quantitative understanding of long‐term population‐level effects. Here we demonstrate the framework's general applicability to other marine and terrestrial systems and show how it can support integrated modeling of the proximate and ultimate mechanisms that regulate trait‐mediated, indirect interactions in ecological communities, that is, the nonconsumptive effects of a predator or stressor on a species' behavior, physiology, or life history.

## 1 INTRODUCTION

The nonlethal effects of disturbance, which we define as a deviation in an animal's physiology or behavior from patterns occurring in the absence of predators or humans (Frid & Dill, 2002), can strongly affect wildlife populations (Lima, 1998). Understanding the population‐level repercussions of these changes in individual behavior and physiology is part of a more comprehensive ecological challenge: the quantification of trait‐mediated indirect effects (Werner & Peacor, 2003), also referred to as nonconsumptive effects (Peckarsky et al., 2008), on ecological interactions. Under this paradigm, a predator or other stressor can affect a species directly via the removal of individuals and alteration of population density (the consumptive or lethal effects), and indirectly by inducing changes in morphology, physiology, behavior, or life history (i.e., the species' traits) that reduce the risk of predation (Schmitz, Krivan, & Ovadia, 2004). Trait‐mediated indirect interactions can, in turn, lead to cascading effects on other components of the ecological community (Peckarsky et al., 2008; Ripple & Beschta, 2012). Characterizing these processes requires an integrative approach that can scale the responses of individual animals to demographic effects in the context of their energy balance and the species' life history (Middleton et al., 2013).

Because the responses of animals to many anthropogenic stimuli are similar to their responses to predation risk (Beale & Monaghan, 2004; Frid & Dill, 2002), the evaluation and management of human activities that have nonlethal effects on wildlife can be framed in this wider context. Assessing the population consequences of disturbance (PCoD) has been a long‐term goal for ecologists, decision makers, and managers and is currently a requirement for most environmental impact assessments under European Union (European Habitats Directive 92/43/EEC) and United States (Marine Mammal Protection Act, 16 U.S.C. §§ 1361 *et seq*.) legislation (King et al., 2015). However, comprehensive assessments of the effects of disturbance are rarely undertaken because of a lack of relevant data and because permit and policy decisions about disturbance must be made within strict timelines. In addition to these constraints, the theoretical understanding and the empirical and analytical methods needed to evaluate these long‐term consequences are often not available. As a result, management decisions have been generally based on evidence of behavioral responses to disturbance, although such responses may have no population‐level effect (Christiansen & Lusseau, 2015). Conversely, the absence of an obvious behavioral response does not rule out a population‐level effect (Gill, Norris, & Sutherland, 2001). Given the increasing expansion of activities that can disturb wildlife, quantitatively linking disturbance to population dynamics is a major objective for modern conservation (Gill et al., 2001). A mechanistic understanding of the processes by which disturbance affects populations is especially useful for long‐lived, wide‐ranging species, for which empirical data are often collected over relatively small spatial and temporal scales (National Research Council, 2005).

**Figure 1:**
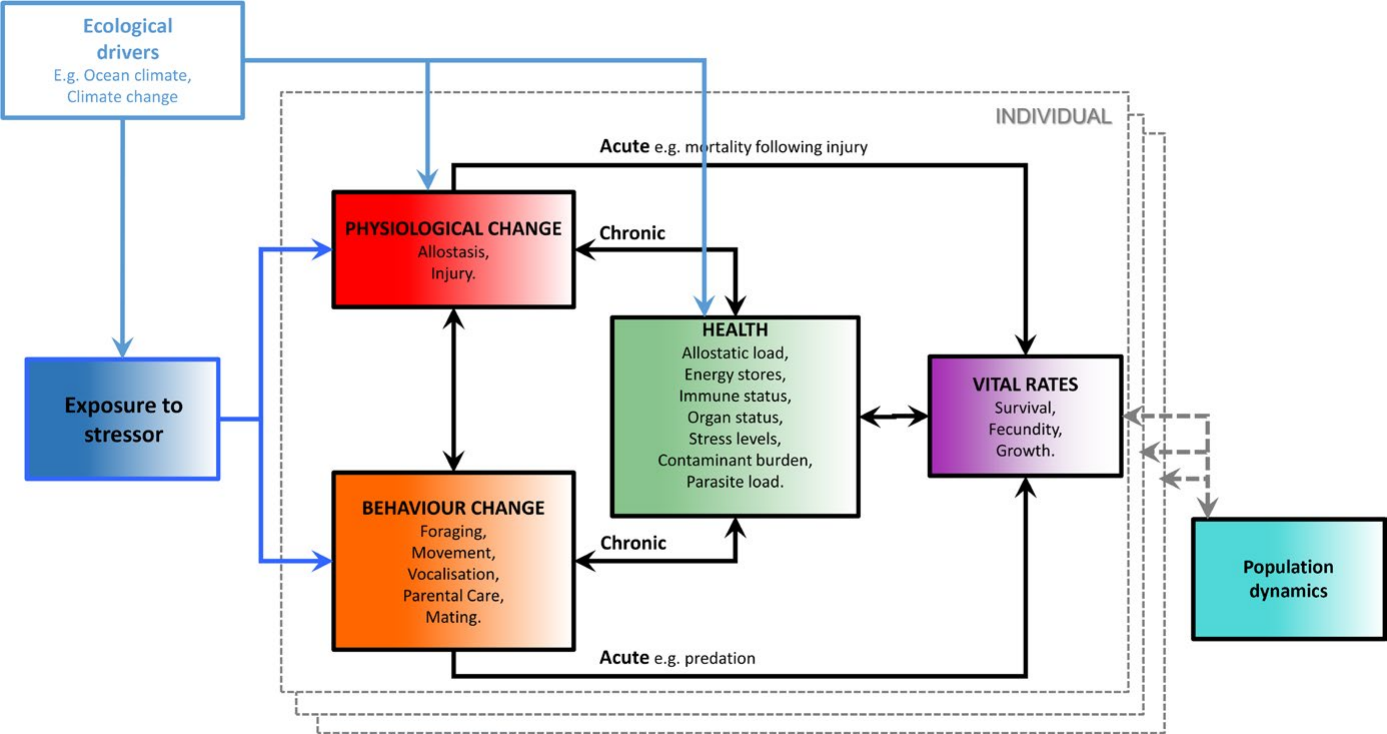
The Population Consequences of Disturbance (PCoD) conceptual framework, modified from National Academies (2017). The boxes within the dashed gray boundary line represent the effects of exposure to a stressor and a range of ecological drivers on the vital rates of an individual animal. The effects are then integrated across all individuals in the population to project their effects on the population's dynamics

Groups established by the National Research Council of the US National Academies and the US Office of Naval Research have addressed ways of modeling the population‐level effects of disturbance on marine mammals (National Academies, 2017; National Research Council, 2005; New et al., 2014). Their efforts led to the development of a conceptual framework that summarizes the functional links among processes (Figure 1). The underlying concept is that disturbance‐induced changes in behavior or physiology affect fitness through individuals' health and vital rates (survival, reproductive success, and growth rate, the latter affecting age at first breeding). The population‐level consequences of changes in individual fitness depend on what proportion of the population is affected, which in turn determines the distribution of fitness‐related traits in the population. The framework provides a way to quantify four phenomena: (a) the physiological and behavioral changes that occur as the result of exposure to a particular stressor, (b) the acute effects of these physiological and behavioral responses on individual vital rates, and their chronic effects via individual health, defined by New et al. (2014) as all internal factors that affect fitness or homeostasis, (c) the way in which changes in health may affect individuals' vital rates, and (d) how changes in individual vital rates may affect population dynamics.

**Figure 2:**
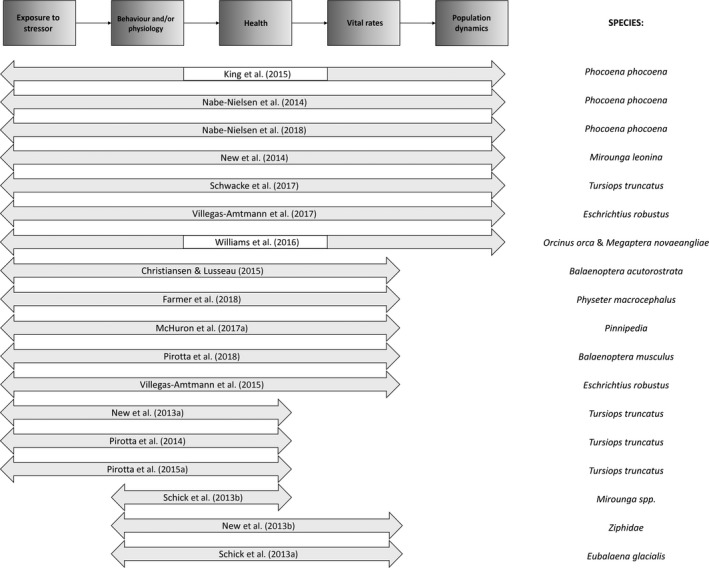
Studies investigating the Population Consequences of Disturbance (PCoD) on marine mammals, updated from Nowacek, Christiansen, Bejder, Goldbogen, and Friedlaender (2016). The arrows indicate the functional steps of the framework (simplified on top) that were included in each study. White gaps in the arrows indicate studies that evaluated the link between behavior and vital rates directly, without estimating health

Thirteen years after the first published formalization of this framework (National Research Council, 2005), we review its applications to marine mammal populations. We first discuss the assessment of the exposure levels of individuals in a population, and then review the approaches that have been used to model each of the functional links in the framework (Figure 2). Our synthesis will help reconcile the marine mammal literature with studies on other taxa that have quantified the effects of anthropogenic disturbance on vital rates and population dynamics (e.g., Broekhuis, 2018; Coetzee & Chown, 2016; Green, Johnson‐Ulrich, Couraud, & Holekamp, 2018; Kight & Swaddle, 2007; McClung, Seddon, Massaro, & Setiawan, 2004; Wood, Stillman, & Goss‐Custard, 2015). We also provide a decision tree that can guide the selection of the most applicable PCoD modeling approach, given the information available.

## 2 ESTIMATING LEVELS OF EXPOSURE IN THE POPULATION

Estimating the population‐level consequences of individuals' responses to disturbance requires information on the proportion of the population that is exposed to the stressor, and the aggregate exposure of each individual (i.e., the total duration and intensity of exposure to the stressor during a given period). In this context, the stressor corresponds to an anthropogenic source of disturbance. The spatial and temporal overlap between the stressor and the focal animals determines the probability of exposure. This overlap is influenced by the patterns by which the stressor is produced at the source and propagates through the environment (Merchant, Faulkner, & Martinez, 2018) and the animals' residence time in the area where exposure may occur (Costa, Hückstädt, et al., 2016). Residence time is determined *inter alia* by the size of individual home ranges, the motivation underlying the use of the area of interest (e.g., whether the area contains foraging patches or is used solely for transit), and any migratory behavior. In some cases, these factors may result in the lack of any geographical or temporal overlap between a population and a stressor, obviating the need to assess population effects.

We believe that evaluating the spatial and temporal overlap between a population's range and the distribution of stressors on the basis of density maps derived from the results of dedicated or historical surveys (Ellison et al., 2016; Hammond et al., 2002) should be a routine component of environmental impact assessments. Telemetry data can provide information on the patterns of repeated exposure for specific individuals (Costa et al., 2003; Falcone et al., 2017; Jones et al., 2017; Madsen et al., 2006; Pirotta, New, & Marcoux, 2018), and photographic identification (e.g., Calambokidis, Barlow, Ford, Chandler, & Douglas, 2009) can be used to estimate exposure risks for regularly monitored populations (Christiansen, Bertulli, Rasmussen, & Lusseau, 2015; Pirotta, Thompson, Cheney, Donovan, & Lusseau, 2015). In alternative, some studies examined the consequences of exposing all individuals in a population to the same amount of disturbance (Braithwaite, Meeuwig, & Hipsey, 2015; New et al., 2014; Villegas‐Amtmann, Schwarz, Gailey, Sychenko, & Costa, 2017; Villegas‐Amtmann, Schwarz, Sumich, & Costa, 2015).

## 3 EFFECT OF EXPOSURE ON PHYSIOLOGY AND BEHAVIOR

The initial step in implementing the PCoD framework (Figure 1) is the quantification of the physiological and behavioral responses of individuals to a known or potential stressor. Controlled exposure experiments (Harris et al., 2018) have used electronic loggers to assess changes in the movement and vocalizations of marine mammals exposed to military sonar and air guns used for seismic surveys (Dunlop et al., 2013; Wensveen et al., 2017). Loggers have also been applied to monitor marine mammal responses to actual disturbance events; for example, of Cuvier's beaked whales (*Ziphius cavirostris*) to sonar exercises (Falcone et al., 2017) as well as of harbor seals (*Phoca vitulina*) to pile driving for wind farm construction (Russell et al., 2016) and to pedestrian and vessel approaches at their haul‐outs (Andersen, Teilmann, Dietz, Schmidt, & Miller, 2014). Visual observations have been used to quantify activity budgets and estimate changes in behaviors such as resting or foraging in the presence of other human activities, such as whale watching (e.g., Christiansen, Rasmussen, & Lusseau, 2013; Lusseau, 2003; New et al., 2015; Williams, Trites, & Bain, 2002). Visual studies on pinnipeds have also monitored flushing response and return to haul‐out sites (e.g., Cowling, Kirkwood, Boren, Sutherland, & Scarpaci, 2015; Osterrieder, Salgado Kent, & Robinson, 2017). Passive acoustic monitoring techniques offer a more continuous alternative to visual sampling for collecting such data on cetaceans. They have been used to assess the responses of harbor porpoises (*Phocoena phocoena*) to wind farm developments (Brandt, Diederichs, Betke, & Nehls, 2011; Nabe‐Nielsen, Sibly, Tougaard, Teilmann, & Sveegaard, 2014; Nabe‐Nielsen et al., 2018), of Blainville's beaked whales (*Mesoplodon densirostris*) to sonar (Moretti et al., 2014; Tyack et al., 2011), and of bottlenose dolphins (*Tursiops truncatus*) to boat presence (Pirotta, Merchant, Thompson, Barton, & Lusseau, 2015). In the absence of empirical data, behavioral responses have been extrapolated from better‐studied species or assumed, often in terms of the number of lost foraging days (King et al., 2015; New et al., 2014; Villegas‐Amtmann et al., 2015, 2017). Most studies (e.g., Williams, Lusseau, & Hammond, 2006) have evaluated the decrease in energy intake due to the observed behavioral responses. However, there have been efforts to quantify the change in energy expenditure associated with avoidance responses (Braithwaite et al., 2015; Christiansen, Rasmussen, & Lusseau, 2014; Miller et al., 2009; Williams, Blackwell, Richter, Sinding, & Heide‐Jørgensen, 2017; Williams, Kendall, et al., 2017). Measuring physiological responses to disturbance is more challenging than measuring behavioral responses, and may require the analysis of tissue, exhalations, or feces from wild animals (Hogg et al., 2009; Rolland et al., 2012), dedicated physiological tags (Karpovich, Skinner, Mondragon, & Blundell, 2015; Williams, Blackwell, et al., 2017; Wilson, Wikelski, Wilson, & Cooke, 2015), or experiments in captivity (Kvadsheim, Sevaldsen, Folkow, & Blix, 2010; Miksis et al., 2001; Thomas, Kastelein, & Awbrey, 1990). Due to these limitations, most applications of the PCoD framework have not modeled the physiological consequences of disturbance explicitly.

## 4 EFFECT OF BEHAVIORAL AND PHYSIOLOGICAL CHANGES ON HEALTH

Some behavioral or physiological changes can have acute effects on individuals' vital rates, for example, by changing their predation risk or because injury directly affects their survival probability (Hooker et al., 2012). However, such changes can also affect vital rates indirectly by impairing an individual's health. Modeling health explicitly provides the mechanistic link scaling individual responses to demographic effects that is required for the assessment of trait‐mediated indirect interactions (Middleton et al., 2013). Although an individual's health encompasses many aspects of its physiology (for example, immune status, stress levels, and contaminant and parasite load, Pettis et al., 2017), most PCoD applications have used an individual's energy stores (that is, its body condition) as the measure of health. For example, New et al. (2014) and Schick, New, et al. (2013) examined the relation between foraging activity and energy stores (estimated from changes in buoyancy) of female southern elephant seals (*Mirounga leonina*) over the course of a foraging trip. Other applications have inferred changes in energy stores from models of foraging activity that either treat energy explicitly using a bioenergetic approach (Beltran, Testa, & Burns, 2017; Christiansen & Lusseau, 2015; Farmer, Noren, Fougères, Machernis, & Baker, 2018; McHuron, Costa, Schwarz, & Mangel, 2017; McHuron, Mangel, Schwarz, & Costa, 2017; Noren, 2011; Pirotta, Mangel, et al., 2018; Villegas‐Amtmann et al., 2015, 2017) or use an arbitrarily scaled energy metric that represents an underlying motivational state (Nabe‐Nielsen et al., 2014, 2018; New, Harwood, et al., 2013; Pirotta, Harwood, et al., 2015; Pirotta, New, Harwood, & Lusseau, 2014). Although technologies that can measure the morphometrics of individuals remotely may make it easier to estimate changes in body condition directly (e.g., Christiansen, Dujon, Sprogis, Arnould, & Bejder, 2016; Miller, Best, Perryman, Baumgartner, & Moore, 2012), extensive health assessment in cetaceans will probably remain limited to a few closely monitored coastal populations, due to logistical constraints (Wells et al., 2004). In contrast, some pinniped populations can be regularly accessed to measure the variation in body condition and health among individuals (e.g., McDonald, Crocker, Burns, & Costa, 2008; McMahon, Harcourt, Burton, Daniel, & Hindell, 2017; Shero, Krotz, Costa, Avery, & Burns, 2015; Wheatley, Bradshaw, Davis, Harcourt, & Hindell, 2006). However, even when such assessments are possible, establishing the cause of observed changes in health is challenging.

## 5 EFFECT OF VARIATIONS IN HEALTH ON VITAL RATES

For most species, few empirical data are available to quantify the relation between an individual's health and its vital rates. New et al. (2014) and Costa, Schwarz, et al. (2016) used empirical data on the relation between a female elephant seal's energy stores at the start of lactation and the weaning mass of her pup, which affects the pup's survival probability (McMahon, Burton, & Bester, 2000, 2003), as the basis for the relation between health and reproductive success. Schick, Kraus, et al. (2013) and Rolland et al. (2016) used state‐space models linking the health of individual North Atlantic right whales (*Eubalaena glacialis*), obtained from the integration of multiple photographic assessments, to their survival and fertility. Schwacke et al. (2017) used a respiratory metric of health to quantify the effect of the *Deepwater Horizon* oil spill on vital rates of bottlenose dolphins in the Gulf of Mexico. In the absence of a direct estimate of calf survival, Christiansen and Lusseau (2015) used the fetal length of minke whales (*Balaenoptera acutorostrata*) as a proxy, and investigated how fetal length was associated with female body condition (Christiansen, Víkingsson, Rasmussen, & Lusseau, 2014). All other PCoD studies of marine mammals have assumed a simple relationship between various health metrics and vital rates (McHuron, Costa, et al., 2017; Nabe‐Nielsen et al., 2014, 2018; Pirotta, Mangel, et al., 2018; Villegas‐Amtmann et al., 2015, 2017).

## 6 A DIRECT LINK BETWEEN EXPOSURE AND VITAL RATES

Few monitoring programs collect information on the changes in individual health and vital rates that may result from behavioral responses to disturbance. In situations where a management decision is needed and this information is not available, a pragmatic alternative is to use a single function to link behavioral responses directly to vital rates. This has been referred to as an interim PCoD approach (King et al., 2015) because this function should be replaced with one based on empirically derived values as soon as they become available. In some of these cases, structured elicitation of information from multiple experts (known as expert elicitation) can provide both estimates of the appropriate parameters and a measure of the associated uncertainty (King et al., 2015; Martin et al., 2012; Oedekoven, Fleishman, Hamilton, Clark, & Schick, 2015). In alternative, the measured effect of changes in prey availability can be used as a proxy for the relation between energy intake and vital rates (Williams, Thomas, Ashe, Clark, & Hammond, 2016). The latter requires the assumption that a reduction in foraging time resulting from disturbance is equivalent to a reduction in the availability of prey.

## 7 MODELING THE EFFECT OF VITAL RATES ON POPULATION DYNAMICS

The final step in the PCoD conceptual model is the propagation of changes in individuals' vital rates to the population. It is beyond the scope of this study to review methods for modeling the dynamics of wildlife populations. Here, we describe the types of population models that have been used, or could be used, to estimate the population‐level effects of disturbance. They lie along a continuum from treating all animals in a population as identical to considering all animals as unique individuals that are followed from birth to death (i.e., individual‐ or agent‐based models [IBMs]).

Because traditional matrix models (Caswell, 2001) are formulated in discrete time, they generally have a birth‐pulse structure. In this structure, all births and deaths are assumed to occur at the same moment in time, which usually corresponds to the transition from one age class to the next, and all individuals within a class are treated as identical. However, classes may be subdivided to reflect the different vital rates of disturbed and undisturbed animals (King et al., 2015). Most PCoD applications have used a simple Leslie matrix to predict the trajectory of a population under different scenarios of anthropogenic disturbance (King et al., 2015; New et al., 2014; Schwacke et al., 2017). Matrix models historically assumed that vital rates are simply a function of an individual's age or stage, but integral projection models (IPMs) account for the additional effects of continuously varying traits (such as physical size) on vital rates (Ellner & Rees, 2006). In principle, a continuous measure of health or the amount of disturbance experienced by different individuals could be modeled as a fitness‐related trait (Coulson, 2012). However, because IPMs do not assign traits to specific individuals, individuals are still not consistently followed over time, and models are formulated in discrete time.

In reality, survival and reproduction are affected by an individual's physiological status and behavior in a complex manner (Houston & McNamara, 1999), and changes induced by disturbance can affect vital rates from the moment at which disturbance occurs. Continuous‐time life‐history models (De Roos, 2008) could therefore be more appropriate for estimating the population‐level effects of disturbance. Although continuous‐time life‐history models also assume individuals are identical, they can readily be structured into multiple classes (De Roos, Galic, & Heesterbeek, 2009).

IBMs follow simulated individuals throughout their life, allowing for explicit modeling of individual heterogeneity and environmental stochasticity in quasi‐continuous time (Grimm & Railsback, 2013). Some PCoD applications developed IBMs that simulate individuals moving, accessing prey, and accumulating energy stores to sustain survival and reproduction (New, Harwood, et al., 2013; Pirotta, Harwood, et al., 2015; Pirotta et al., 2014; Villegas‐Amtmann et al., 2015). However, only three studies (Nabe‐Nielsen et al., 2014, 2018; Villegas‐Amtmann et al., 2017) have used IBMs to predict the dynamics of a population over time. Although IBMs require considerable data, they are extremely flexible. In addition, simple models with sufficient realism can often be constructed on the basis of a relatively small amount of empirical information. Unknown parameters may, as an interim measure, be extrapolated from a species with a comparable life history (Sibly et al., 2013), as long as their influence on the model's outcome is explicitly quantified and acknowledged, for example, using sensitivity analysis. Model parameters then can be optimized with standard calibration techniques (Grimm & Railsback, 2013), or fitted to data with Bayesian inference (Kattwinkel & Reichert, 2017). IBMs can also be implemented via stochastic dynamic programming (Mangel & Clark, 1988) and used to estimate optimal behavior given estimates of state variables over time. The ability of this approach to forecast population‐level effects of disturbance was demonstrated for pinnipeds by McHuron, Costa, et al. (2017) and for Eastern North Pacific blue whales (*Balaenoptera musculus*) by Pirotta, Mangel, et al. (2018), whereas Klaassen, Bauer, Madsen, and Tombre (2006) used stochastic dynamic programming to quantify the effects of disturbance on the survival of Svalbard pink‐footed geese (*Anser brachyrhynchus*).

## 8 CHOOSING A MODEL STRUCTURE

**Figure 3:**
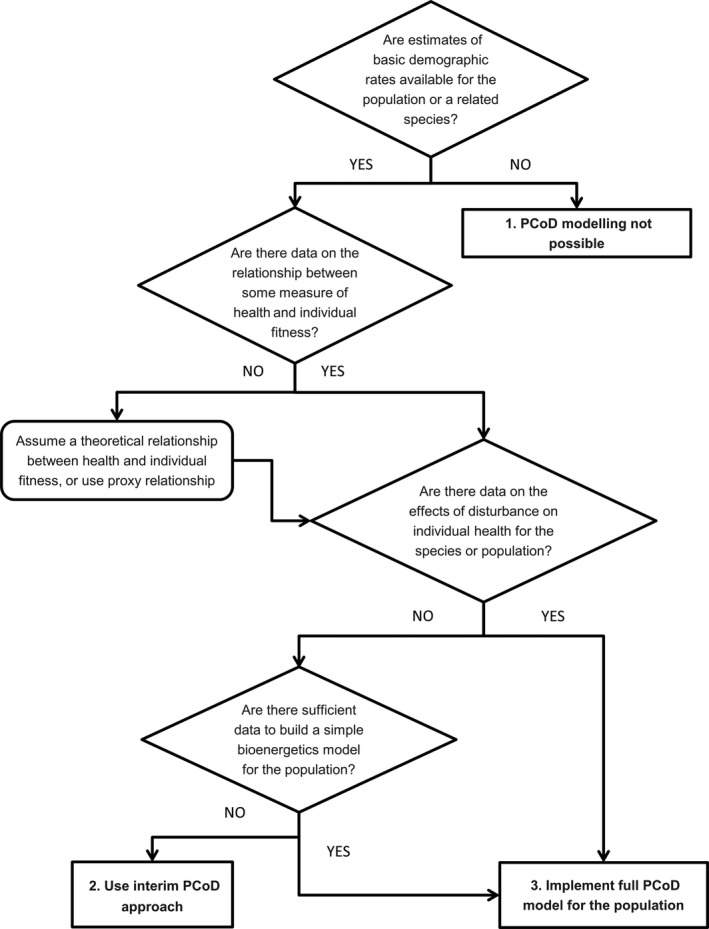
Decision tree to guide selection of the most suitable Population Consequences of Disturbance (PCoD) model for a given population, given data availability. Decision points are represented by diamonds, and possible outcomes by rectangles

In most situations, selection of a model structure to forecast the population‐level effects of disturbance is likely to be driven by data availability (Figure 3). No PCoD model to date has been fully parameterized with empirical data. Even models that encompass the chain from exposure to population dynamics (King et al., 2015; Nabe‐Nielsen et al., 2014, 2018; New et al., 2014; Villegas‐Amtmann et al., 2017; Williams et al., 2016) have used data extrapolated from other species, expert judgments, proxy relations, or informed assumptions.

The first step in choosing a model is to evaluate the availability of estimates of basic demographic parameters, such as vital rates, population growth rate, or age at first breeding or maturity. Precise estimates of these parameters are not essential, and missing values can be inferred if reliable estimates of other demographic attributes are available. If no demographic information is available for the target population or a related species, PCoD modeling is not possible (outcome 1 in Figure 3), but insights into a population's propensity for PCoD can be obtained from first principles using prospective approaches (Nattrass & Lusseau, 2016). Although some demographic parameters are emergent properties of bioenergetic models (see below), estimates of these parameters are needed to calibrate the models and validate their predictions.

The second step is to assess evidence of empirical relations between measures of individual health and fitness. For example, New et al. (2014) estimated a relation between pup survival and total body lipid of adult female elephant seals. If no empirical information is available, a simple, theoretical relation between an individual's health and its subsequent survival, fecundity, or growth can be assumed (New, Moretti, Hooker, Costa, & Simmons, 2013). Typically, such relations are hyperbolic (Nabe‐Nielsen et al., 2014) or sigmoidal (McHuron, Costa, et al., 2017).

The third step is to investigate whether there are empirical data on the relation between behavioral change and individual health. If such information is unavailable, bioenergetic models can be used to examine the potential effects of lost foraging opportunities on an individual's health, as measured by its energy stores (McHuron, Costa, et al., 2017; Nabe‐Nielsen et al., 2014, 2018; Pirotta, Mangel, et al., 2018; Villegas‐Amtmann et al., 2015, 2017). This allows construction of a full PCoD model for the population (outcome 3 in Figure 3), as in New et al. (2014). The basic data required to construct such bioenergetic models are duration of gestation and lactation, a growth curve to predict mass at different ages, and an estimate of field metabolic rate (Costa & Maresh, 2017; Villegas‐Amtmann et al., 2015). Ideally, these data should be collected from the target species or population, but they can be derived theoretically or from related species. The resulting models can be calibrated using information on the demography of the population, such as the ratio of calves to mature females. Expert elicitation can be used to fill some knowledge gaps, for example, to estimate the level of energy stores below which starvation mortality may occur, or to establish whether the rate of milk transfer is determined by the offspring or the mother.

If there is insufficient information to develop a bioenergetic model, expert elicitation can be used to estimate a direct relation between behavioral change and vital rates. This is represented by outcome 2 of the decision tree (Figure 3): the use of an interim PCoD approach (King et al., 2015).

## 9 INCORPORATING UNCERTAINTY

Whichever model is chosen, it is necessary to quantify uncertainty at all stages of modeling (Harwood & Stokes, 2003; Milner‐Gulland & Shea, 2017). Uncertainty arises through the precise choice of model parameterization, the specification of input parameter values, environmental stochasticity, and variation among individuals. Incorporation and propagation of these uncertainties vary among the modeling approaches described above. For example, in IBMs, it is possible to simulate from distributions on input parameters, and to include environmental stochasticity and variation among individuals in the simulation (Grimm & Railsback, 2013). To incorporate model uncertainty, simulations can sample from alternative parameterizations, although this rarely is done in practice. By contrast, life‐history models are intrinsically deterministic, although it is possible to repeat the modeling with different inputs. In all cases where uncertainty is not explicitly incorporated into the modeling, we suggest that a sensitivity analysis be performed post hoc to determine the robustness of conclusions to plausible violation of model assumptions and variation in the inputs. Uncertainty in the estimated population consequence ultimately can be reported as a distribution of potential outcomes. This will allow the precautionary principle to be applied if the results are used to make management decisions.

## 10 APPLICATIONS OF PCoD MODELS

Since its formulation (National Research Council, 2005), the PCoD conceptual model has served as a common framework for examining the potential effects of nonlethal human disturbance on marine mammal populations, accounting for the uncertainties associated with each step in the process. Use of this model has changed the focus of the scientific discussion from establishing subjective thresholds of acceptable behavioral change to quantifying long‐term, population‐level effects (National Academies, 2017).

Real‐world applications of the PCoD framework in the last decade used a range of modeling approaches to translate the conceptual model into a mathematical structure, and highlighted challenges and data gaps (Figure 2). The effects of disturbance on population size predicted in these studies were generally too small for short‐term detection with conventional methods of abundance estimation (Taylor, Martinez, Gerrodette, Barlow, & Hrovat, 2007). Yet these effects could have substantial medium‐term effects on population status.

To remain tractable, most PCoD models to date considered one disturbance source or scenario in isolation. However, multiple sources of disturbance are likely to occur in an area at any given time, together with other, concurrent environmental and ecological processes. Attributing causation to a single stressor and developing mitigation measures therefore is challenging in practice. Accordingly, the PCoD framework recently was expanded to incorporate the cumulative effects of multiple stressors and ecological drivers (National Academies, 2017).

Although models of the population consequences of anthropogenic disturbance have been developed to assess the effects of expanding human activities in the ocean on marine mammal populations, the PCoD framework is relevant for other marine and terrestrial taxa. The literature on the effects of human disturbance on wildlife behavior is extensive (e.g., Blumstein, Fernández‐Juricic, Zollner, & Garity, 2005; Stankowich, 2008). Moreover, many studies have linked changes in behavior deriving from interactions with humans to the survival and reproductive success of individuals (e.g., Broekhuis, 2018; Dussault, Pinard, Ouellet, Courtois, & Fortin, 2012; Ellenberg, Mattern, Seddon, & Jorquera, 2006; Giese, 1996; Gosselin, Zedrosser, Swenson, & Pelletier, 2014; Kerley et al., 2002; Kight & Swaddle, 2007; McClung et al., 2004; Rodriguez‐Prieto & Fernandez‐Juricic, 2005), and some have quantified the long‐term effects on population dynamics (e.g., Coetzee & Chown, 2016; Green et al., 2018; Iverson, Converse, Smith, & Valiulis, 2006; Wood et al., 2015). These studies could be incorporated into the unifying framework we describe here to model the effects of many forms of nonlethal anthropogenic disturbance.

There is interest in integrating proximate (mechanisms and functions) and ultimate (adaptation and fitness value) aspects of behavior into conservation (Cooke et al., 2014; Sutherland, 1998). Knowledge of the physiological mechanisms of an animal's interaction with its environment is also relevant to conservation (Wikelski & Cooke, 2006). The PCoD approach provides a means for investigating the physiological and the behavioral drivers of an individual's response to human disturbance, and therefore a population's viability (Cooke et al., 2014). Linking behavioral and physiological changes to demography also facilitates prediction of the effects of climate change on wildlife populations (e.g., Desprez, Jenouvrier, Barbraud, Delord, & Weimerskirch, 2018; Pagano et al., 2018; Weimerskirch, 2018). More generally, the nonlethal effects of human disturbance and environmental change and their repercussions at a population level can be viewed as examples of the fundamental processes regulating trait‐mediated, indirect ecological interactions (Ripple & Beschta, 2012; Werner & Peacor, 2003). Disentangling consumptive and nonconsumptive effects requires an understanding of the interactions among the individual, population, and community levels (Schmitz et al., 2004), and integrative approaches are necessary to link individual behavior, energy balance, and life history and to investigate demographic effects (Middleton et al., 2013). The PCoD approach therefore could offer a formal framework for the investigation of the proximate mechanisms of these and other ecological phenomena that operate via changes at the individual level. Experience already gained from application of PCoD models to marine mammal populations provides practical guidance for model development and data collection (Fleishman et al., 2016).

## CONFLICT OF INTEREST

None declared.

## AUTHORS' CONTRIBUTIONS

All authors contributed to discussions that supported the development of the framework and led to the present review of its applications. EP and JH led the writing of the manuscript. All authors contributed critically to the drafts and gave final approval for publication.

## DATA ACCESSIBILITY

No data are associated with this manuscript.
